# A Case of Unilateral Involuntary Movements Treated with Procaine Amide

**Published:** 1961-07

**Authors:** W. Cullen, A. Leitch

**Affiliations:** Senior Specialist in Psychiatry, R.N.; Barrow Hospital, Barrow Gurney, near Bristol


### A CASE OF UNILATERAL INVOLUNTARY MOVEMENTS TREATED WITH PROCAINE AMIDE

BY

W. CULLEN, M.B., CH.B., SURGEON-COMMANDER R.N.

(*Senior Specialist in Psychiatry, R.N.*)

AND A. LEITCH, M.D., D.P.M.

*Barrow Hospital, Barrow Gurney, near Bristol*

Although there have been several reports on the treatment of Huntingdon's chorea with procaine amide (Bruyn, 1958; Merskey, 1958; Lazarte *et al.* 1955; DeMeyer and Dyken, 1954; Goldman, 1952), it is felt that the following case is worthy of record.

### CASE HISTORY

The patient, a married man of 73 years of age, was admitted to hospital in January, 1960, from his home, with a history of depression, dyspepsia and involuntary choreiform movements of the left side of his body.

The previous history was that of a healthy, active, hardworking and stable man who had no history of previous mental or nervous illness. He had worked regularly as a platelayer on the railway for some 47 years till his retirement at 65 years of age; since then till the onset of his illness he had worked regularly as a jobbing gardener. He was happily married and denied any significant family or personal history.

He had been well until six weeks prior to admission. Then he had suffered from an acute attack of bronchitis as he expected to do each winter. He made a slow recovery from the bronchitis and three weeks later he complained of nausea, dyspepsia, and involuntary movements in his left arm and leg.

His condition remained stationary until three days prior to admission, when the movements suddenly became much more severe, so severe in fact that he could not eat or sleep, and tended to fall out of bed. He became very lowspirited with these symptoms and was admitted to hospital for investigation.

In hospital, clinical examination showed him to be an old man with a dry, inelastic skin, who had lost weight. Examination of the chest was negative apart from a few scattered rhonchi in both lung fields. The heart was of normal size and the sounds were well heard, regular in amplitude and rhythm; B.P. was 130/76 mm. Hg. The peripheral vascular system was obviously markedly arteriosclerotic, with the vessels tortuous and cordlike.

The abdomen showed no abnormal findings on examination. Urinary examination showed a slight trace of protein in the urine.

Haematology was reported within normal limits. X-ray of chest showed partial collapse at the left base. Blood W.R. and Kahn tests were negative.

Examination of the C.N.S. was surprisingly negative, apart from the obvious and gross choreiform movements of the left limbs in which the neck and head were also involved. Tendon and superficial reflexes were brisk and equal on both sides; the plantar reflex was flexor, and equal on both sides. Cranial nerves were intact and the fundi showed no changes beyond those to be expected in a man of his age. Co-ordination in the unffected limbs was good. No disturbance of sensation was apparent.

From the psychiatric point of view, the only evidence of disturbance was his mood disorder, which was one of fairly severe depression, reactive to his physical disability. Psychological testing gave him an I.Q. of 93 (average) and the psychologists' conclusion was that the patient was a man of barely average intelligence who displayed no abnormal intellectual deterioration relative to his advanced age.

An EEG examination was also carried out; the result was reported as follows: "No abnormal rhythms were noted nor were any obvious asymmetries observed".

It was concluded, after the investigations noted above and assessment of the findings, that this was a case of cerebral arteriosclerosis with unilateral left-sided involuntary movements presenting as the major clinical features, due to a focal subcortical lesion, probably involving the subthalamic nucleus, and resulting in the hemiballismus syndrome.

Initially the patient was treated by general hospital measures and sedation with sodium amytal. He made no material response to this therapy and treatment with procaine amide was instituted. The initial dose was 0·5 gm. t.d.s. and this was increased after about one week to 1 gm. t.d.s. On this treatment, the choreiform movements quickly subsided, becoming noticeable only when he was tired at the end of the day and then only in the left leg and foot. The depressive symptoms also remitted steadily as his physical condition improved.

Eventually, almost two months after his admission to hospital, he was discharged to his home as "cheerful and free from depression—involuntary movements minimal".

He has been seen again as an out-patient up to six months after his discharge from hospital; he has maintained the improvement he made in hospital and is now a cheerful and alert man who complains only of involuntary movements in the left leg and foot at the end of the day or after unusual exertion.

### DISCUSSION

Martin (1927 and 1928) described a case of hemichorea resulting from a local lesion of the brain—the syndrome of the body of Luys—in a man of 62 years of age, formerly a seaman, with a previous history of hypertension (systolic B.P. 190 mm. Hg) with a positive Wassermann reaction of the blood and markedly atheromatous arteries but no other relevant history. The onset of the illness was acute; the patient suffered from gross involuntary movements of the right side of the body which ceased during sleep; his speech became slurred, swallowing was performed by spasmodic gulps; breathing was irregular and sometimes difficult owing to the movements of throat, chest and abdomen. In spite of the striking clinical picture, neurological examination revealed few abnormalities; voluntary power was normal in all the limbs and there was no sensory loss to pin prick or light touch. Sense of position was normal and pointing tests, including the Barany tests, were accurately performed. Vibration sense was normal; tendon jerks were present in both arms and about equal on the two sides, those in the right arm being if anything less; the knee jerk was less on the affected side; ankle jerks were approximately equal; the plantar responses were flexor throughout the illness. The optic discs were normal and pupils were approximately equal but neither was quite regular; both reacted to light and accommodation. Except for the abnormal movements in the fact, throat and tongue, the other cranial nerves were normal. After an illness of 20 days' duration the patient died of bronchopneumonia. Post-mortem examination showed a recent haemorrhage destroying the corpus Luysii of the left side.

In the discussion of this case, Martin goes on to consider other cases previously described. He finds that they are infrequent; he is able to report on 12 cases dating from 1883 to 1925; he records that in all the well-developed cases death has resulted in a few weeks and that the commonest cause of death was bronchopneumonia.

Whittier (1947) reviewed the literature on this illness, revealing a total of 30 cases of hemiballism with localized pathological changes in the subthalamic nucleus (corpus Luysii: nucleus hypothalamicus). In his analysis of this series, he described both the chronology of the cases and the age distribution of the patients (Tables 1 and 2).

TABLE 1

| Decade | No. of cases |
|---|---|
| 1880–89 | 2 |
| 1880–99 | 0 |
| 1900–09 | 1 |
| 1910–19 | 3 |
| 1920–29 | 1 |
| 1930–39 | 14 |
| 1940–47 | 2 |

TABLE 2

| Age Group | No. of Cases |
|---|---|
| 40–49 | 1 |
| 50–59 | 8 |
| 60–69 | 10 |
| 70–79 | 8 |
| 80–90 | 2 |
| Not stated | 1 |

He reached the following conclusions:

(1) Ballism is a distinct form of involuntary movement characterized by continuous violent activity of the appendicular musculature, such that limbs are flung about.

(2) Hemiballism, and sometimes monoballism of the upper extremity, is the apparently inevitable symptom in man of destruction localised in the contralateral subthalamic nucleus.

(3) There is evidence that interruption of the connections of the subthalamic nucleus may result in hemiballism.

(4) Hemiballism may appear without demonstrable pathologic change in the subthalamic nucleus or its connections, although such cases are relatively rare.

(5) Ballistic movements may appear in a variety of dyskinesias to which the relation of the subthalamic nucleus is as yet obscure. Their similarity to or identity with those of hemiballism following damage to the subthalamic nucleus awaits a more complete and preferably cinematographic comparison.

Russell Brain (1951) describes hemiballismus under the heading of senile chorea and states that the symptoms and prognosis of senile chorea are similar in many respects to those of Huntingdon's chorea except that the age of onset of senile chorea is generally later than that of Huntingdon's variety, and that psychiatric symptoms are less marked. Brain makes no specific reference to treatment of this condition; he infers that no treatment is effective and that the prognosis is hopeless, institutional care being necessary sooner or later in the course of the illness. T allow *et al.*, (1952) are equally pessimistic.

Kinnier Wilson (1955) believed that Kussmaul and Fischer in 1911 were the first to introduce the name "hemiballismus" to describe involuntary movements of extreme violence arising suddenly and involving one side of the body. He refers to both Martin's and Whittier's work, which has already been mentioned in this discussion. He believed that the prognosis is hopeless and that most patients die after a few days or some weeks from increasing exhaustion, cardiac failure or pneumonia. Treatment was again said to be unsatisfactory; sedatives, atropine and stramonium were of little value; and sometimes it might be necessary to tie the affected limb to the patient's side (Bertrand and Garcin, 1933).

Amputation of the affected limb has been performed (Schaller, 1937); paralysis of the brachial plexus by multiple injections of alcohol has also been employed (Kulenkampff, 1938). Central surgery has been used in this condition; Bucy (1944) removed the shoulder and arm area in the precentral gyrus with some of the adjacent frontal lobe; Meyers *et al.* (1950) have advised linear cortico-subcortical section or midbrain pryamidotomy.

The first positive reference to treatment which is effective, without involving surgery with possible serious disablement, seems to have been made by Goldman (1952). He describes an accidental observation: a patient suffering from Huntingdon's chorea received an injection of procaine amide for dental treatment, and was seen to be sitting quietly in the dental chair with minimal choreiform activity after the injection.

This led to a trial of procaine amide by mouth in a total of six patients for the treatment of Huntingdon's chorea. The results were at times dramatic and at time moderate the drug was found to reduce choreiform activity somewhat variably, but, as the author observes "this is the first observation of any useful treatment in such patients".

DeMeyer and Dyken (1954), in a further trial of oral procaine amide in the treatment of Huntingdon's chorea, were unable to detect any objective evidence of improvement in their cases of longstanding chorea. They noted, however, that seven patients reported that they were better. Again, Lazarte *et al.* (1955) reported no improvement in a limited trial of procaine amide hydrochloride. More recently Merskey (1958) has reported in detail on a series of eight patients with Huntingdon's chorea treated with procaine amide. Symptomatic motor benefit was demonstrated in two patients; the other six patients showed no definite signs of improvement or of deterioration. In spite of this seeming lack of response, Merskey believes that procaine amide should be given an adequate trial in all cases of adult chorea; he draws attention to the difficulty of assessing alteration in the frequency of the involuntary movements and to the occurrence of a possible "rebound phenomenon" on ceasing the drug.

Bruyn (1958) reported good results in cases of Huntingdon's chorea with procaine amide, using a dosage of 250 mgm q.i.d., gradually increased to 5 gm daily. When further improvement ceased he added reserpine 0·1 mgm t.d.s. plus 75 mgm pyridoxin daily, with beneficial results.

Phemister (1959) used procaine amide as a muscle relaxant in conditions involving severe muscular hypertonicity and flexor spasms, e.g. following cerebral vascular accidents. He believed that the hypertonicity was a response to the proprioceptive impulses from the muscle spindles. Forrester (1959) contested this, however, and claimed on the basis of experimental evidence that the good results of treatment with procaine amide are due to its interference with the nervous impulses from the gamma fibres which innervate the muscle spindles, thus causing relaxation of the muscle spindles; and that it acts as a cholinergic agent.

The present patient is considered to have presented the typical signs of hemiballismus and was so disturbed by the violent and gross involuntary movements of the left half of his body that he was unable to eat or sleep. He became very depressed as a result and it was clear on his admission to hospital that, if his symptoms of organic nervous dysfunction were not relieved quickly, he would either die of exhaustion and intercurrent infection or he would attempt suicide. Response to procaine amide in this case was quick and substantial, so much so as to justify the description of dramatic.

Side effects have been described by Merskey (1958) including nausea, vomiting, and gastric discomfort. These were not observed in this case. Agranulocytosis, severe hypotensive reactions and ventricular fibrillation have also been described as occasional hazards in treatment with procaine amide; again, none of these was noted in the present case.

### SUMMARY

A case of hemiballism was treated with procaine amide with marked symptomatic relief and with no side effects during treatment.

The nature and incidence of hemiballismus are discussed and the literature available to the authors is reviewed.

Recent developments in the treatment of chorea with procaine amide are reviewed.

### REFERENCES

Bertrand, I. and Garcin, R. (1933). *Rev. Neurol.*, **2**, 820.
Brain, W. Russell (1951). *Diseases of the Nervous System.* Oxford University Press, London.
Bruyn, G. W. (1958). *Fol. psychiat. neerl.*, **61**, 375.

Bucy, P. C. (1944). *The Precentral Motor Cortex*, pp. 361 and 404.
DeMeyer, W. and Dyken, M. (1954). *Amer. J. Med. Sc.*, **228**, 70.
Forrester, T. M. (1959). *Lancet*, **ii**, 913.
Goldman, D. (1952). *Amer. J. Med. Sc.*, **224**, 573.
Kulenkampff, D. (1938). *Zbl. Chir.*, **65**, 2466.
Lazarte, J. A., Baars, C. W. and Pearson, S. S. (1955). *Amer. J. Med. Sc.*, **229**, 676.
Martin, J. P. (1927). *Brain*, **1**, 637.
Martin, J. P. (1928). *Lancet*, **ii**, 315.
Merskey, H. (1958). *J. ment. Sci.*, **104**, 411.
Meyers, R., Sweeney, D. B. and Schwidde, J. T. (1950). *J. Neurol. Psychiat.*, **13**, 115.
Phemister, J. C. (1959). *Lancet*, **ii**, 792.
Schaller, W. F. (1937). *Arch. Neurol. Psychiat.*, **41**, 365.
Tallow, W. F. T., Ardis, J. A. and Bickford, J. A. R. (1952). *Synopsis of Neurology*. John Wright & Sons Ltd., Bristol.
Whittier, J. R. (1947). *Arch. Neurol. Psychiat.*, **58**, 672.
Wilson, S. A. Kinnier (1955). *Neurology* (*ed.* A. Ninian Bruce), 3rd Edition. Butterworth & Co. London.

